# Bacterial flagellin elicits widespread innate immune defense mechanisms, apoptotic signaling, and a sepsis-like systemic inflammatory response in mice

**DOI:** 10.1186/cc9235

**Published:** 2010-08-24

**Authors:** Joëlle Rolli, Noureddine Loukili, Sandra Levrand, Nathalie Rosenblatt-Velin, Stéphanie Rignault-Clerc, Bernard Waeber, François Feihl, Pal Pacher, Lucas Liaudet

**Affiliations:** 1 Department of Intensive Care Medicine, University Hospital Medical Center and Faculty of Biology and Medicine, rue du Bugnon 46, Lausanne 1010, Switzerland; 2 Division of Pathophysiology, Department of Internal Medicine, University Hospital Medical Center and Faculty of Biology and Medicine, Rue du Bugnon 46, Lausanne 1010, Switzerland; 3 Laboratory of Physiologic Studies, National Institute on Alcohol Abuse and Alcoholism, National Institutes of Health, Bethesda, MD 20892-9413, USA

## Abstract

**Introduction:**

Systemic inflammation in sepsis is initiated by interactions between pathogen molecular motifs and specific host receptors, especially toll-like receptors (TLRs). Flagellin is the main flagellar protein of motile microorganisms and is the ligand of TLR5. The distribution of TLR5 and the actions of flagellin at the systemic level have not been established. Therefore, we determined TLR5 expression and the ability of flagellin to trigger prototypical innate immune responses and apoptosis in major organs from mice.

**Methods:**

Male Balb/C mice (*n *= 80) were injected intravenously with 1-5 μg recombinant *Salmonella *flagellin. Plasma and organ samples were obtained after 0.5 to 6 h, for molecular investigations. The expression of TLR5, the activation state of nuclear factor kappa B (NFκB) and mitogen-activated protein kinases (MAPKs) [extracellular related kinase (ERK) and c-jun-NH2 terminal kinase (JNK)], the production of cytokines [tumor necrosis alpha (TNFα), interleukin-1β (IL-1β), interleukin-6 (IL-6), macrophage inhibitory protein-2 (MIP-2) and soluble triggering receptor expressed on myeloid cells (TREM-1)], and the apoptotic cleavage of caspase-3 and its substrate Poly(ADP-ribose) polymerase (PARP) were determined in lung, liver, gut and kidney at different time-points. The time-course of plasma cytokines was evaluated up to 6 h after flagellin.

**Results:**

TLR5 mRNA and protein were constitutively expressed in all organs. In these organs, flagellin elicited a robust activation of NFκB and MAPKs, and induced significant production of the different cytokines evaluated, with slight interorgan variations. Plasma TNFα, IL-6 and MIP-2 disclosed a transient peak, whereas IL-1β and soluble TREM-1 steadily increased over 6 h. Flagellin also triggered a marked cleavage of caspase-3 and PARP in the intestine, pointing to its ability to promote significant apoptosis in this organ.

**Conclusions:**

Bacterial flagellin elicits prototypical innate immune responses in mice, leading to the release of multiple pro-inflammatory cytokines in the lung, small intestine, liver and kidney, and also activates apoptotic signalling in the gut. Therefore, this bacterial protein may represent a critical mediator of systemic inflammation and intestinal barrier failure in sepsis due to flagellated micro-organisms.

## Introduction

Systemic inflammation and multiple organ dysfunction during Gram-negative sepsis have been largely attributed to the activation of innate immune defenses by lipopolysaccharide (LPS) [1]. Accordingly, recent studies showed that strategies interfering with LPS-dependent signaling, including myeloid-differentiation factor-2 [2] and toll-like receptor (TLR) 4 (TLR4) [1] proved beneficial in experimental Gram-negative sepsis. In addition to LPS, most enteric Gram-negative bacteria also release substantial amounts of flagellin, the main structural protein from the bacterial flagellum [3]. Flagellin binds to TLR5 [4] and activates the pro-inflammatory transcription factor nuclear factor (NFκB) in various epithelial cells, endothelial cells and leukocytes *in vitro *(see [3] for review). *In vivo*, the flagellin-TLR5 axis has been associated with the development of cardiovascular collapse [5], acute lung inflammation [6] and inflammatory bowel diseases [7] in mice. Importantly, significant concentration of flagellin circulate in the plasma of human patients with Gram-negative sepsis [6], suggesting that it might represent a significant pro-inflammatory bacterial protein in this setting.

Therefore, the present study was designed to determine the distribution of TLR5 in major organs of mice (lung, liver, kidney and intestine), and to evaluate the ability of these organs to mount an innate immune response to exogenously administered recombinant flagellin. Our main findings indicate that TLR5 is expressed by all the organs examined, and that flagellin elicits prototypical immune signaling in these organs, characterized by the activation of NFκB and mitogen-activated protein kinases (MAPKs), as well as the production of multiple inflammatory cytokines, and also that flagellin initiates proapoptotic responses predominantly in the intestine. Thus, flagellin/TLR5 signaling elicits several mechanisms that are instrumental in the pathophysiology of sepsis, and might therefore represent a novel target for therapeutic intervention.

## Materials and methods

### Administration of flagellin to conscious mice

All procedures conformed to the Swiss laws on the care and use of laboratory animals and were approved by our local ethical committee for animal experimentation. Male BALB/c mice (weighing 23 to 26 g) were injected (tail vein) with recombinant *Salmonella muenchen *flagellin (Calbiochem, San Diego, CA, USA), given at doses of 1 or 5 μg/mouse. Such doses are pathophysiologically and clinically relevant, because free flagellin, at up to several hundred μg/L, is detectable in the plasma of rats with lethal Gram-negative bacteria-induced peritonitis [5], and free flagellin circulates at levels between 2 and 20 μg/L in the blood of patients with Gram-negative sepsis [6]. Flagellin was suspended in a volume of 0.2 ml isotonic saline. Sham animals injected with saline only were used for control purposes. The flagellin preparation was devoid of LPS contamination, as indicated by the Limulus assay (< 0.0003 μg LPS/μg flagellin). At selected time-points (30 minutes to 6 hours), mice were sacrificed by pentobarbital overdose, and the lung, liver, small intestine and kidney were removed for subsequent analyses. Plasma was collected for the measurement of cytokines.

### RNA Isolation, RT-PCR and quantitative real-time PCR

Total RNA from tissues was isolated by TRIzol reagent (Invitrogen, Basel, Switzerland). RNA was reverse-transcribed to cDNA and amplified by PCR (One-step RT-PCR kit from Qiagen, Hombrechtikon, Switzerland). A kit of mouse-specific PCR primers was purchased for TLR5 (R&D Systems, Minneapolis, MN, USA). GAPDH was used for control purposes. The cDNAs were further subjected to quantitative real-time PCR using the SYBR Premix Ex Taq (Takara Bio Inc., Otsu, Shiga, Japan) on a LightCycler Instrument (Roche Applied Science, Rotkreuz, Switzerland). Cycling conditions consisted of an initial denaturating step at 95°C (20 seconds) followed by 45 cycles of a thermal step protocol consisting of 95°C (30 seconds), 60°C (20 seconds). Primer sequences for TLR 5 were 5'-CCA CCG AAG ACT GCG ATG A-3' and 5'-GTG ACC GTG CAC AGG ATG AA-3'. The data obtained for TLR5 were normalized to the level of 18 S (5'-ACT TTT GGG GCC TTC GTG TC-3'; 5'-GCC CAG AGA CTC ATT TCT TCT TG-3'), and expressed in fold augmentation with respect to the expression in the kidney (which had the weakest expression).

### Protein extraction and immunoblot analyses

Tissues were homogenized in ice-cold lysis buffer, and cytoplasmic and nuclear proteins were obtained as described [8]. Proteins (20 to 80 μg) were separated by a standard SDS-PAGE procedure. The following antibodies were used for immunoblotting: anti-inhibitor of kappaB alpha (IκBα), anti-phospho-IκBα, anti-c-jun-NH2 terminal kinase (JNK)1, anti-extracellular related kinase (ERK)1/2 (all from Santa-Cruz Biotechnology, Santa Cruz, CA, USA), anti-phospho-JNK1/2, anti-phospho-ERK1/2, anti-poly(ADP-ribose) polymerase (PARP), anti-caspase-3, anti-cleaved-caspase-3 (all from Cell Signaling, Beverly, MA, USA), anti-β-actin, anti-α-tubulin (Sigma-Aldrich, Basel, Switzerland) and anti-TLR5 (Biovision Inc., Mountain View, CA, USA). The immunoblot signals were visualized using enhanced chemiluminescence (ECL, Amersham Biosciences, Otelfingen, Switzerland). Densitometric analysis was performed using a Personal Densitometer TM (Molecular Dynamics, Sunnyvale, CA, USA) and TotalLab software (TotalLab, Newcastle upon Tyne, UK), as described [9].

### Electromobility shift assay

Electromobility shift assay (EMSA) was performed as described [8], by incubating 10 μg of nuclear proteins with an NFκB oligonucleotide probe (5'-GGCAGTTGAAGGGGACTTTCCCAGG-3') labeled with α-^32^PdCTP, and poly dIdC for 20 minutes. For supershift assays, the nuclear extracts were preincubated for 30 minutes on ice with an anti-p65 or an anti-p50 antibody (both from Cell Signaling, Beverly, MA, USA) before adding the radioactive probe. Samples were resolved on a non-denaturing polyacrylamide gel. Gels were transferred to Whatman 3 M paper (Fisher Scientific, Wohlen, Switzerland), dried under vacuum, and exposed to photographic film at -70°C with intensifying screens.

### Quantification of cytokine production

The tissue concentrations of TNFα, IL-1β, IL-6, macrophage-inhibitory protein (MIP)-2 and soluble triggering receptor expressed on myeloid cells (sTREM-1) were measured in tissue homogenates using commercially available ELISA kits (R&D Systems, Minneapolis, MN, USA) according to manufacturer's protocol. The same cytokines were also measured in plasma.

### Presentation of data and statistical analysis

All graphs summarize the results of at least three independent experiments, and are presented as means ± standard error of the mean. In experiments comparing only two conditions, statistical analysis was performed with Student's *t *test. In experiments using multiple conditions, comparison was performed with analysis of variance. When the F value was significant at the 5% level, further pair-wise comparisons were made between flagellin and control conditions using Dunnett's test. A *P *value less than 0.05 was considered significant.

## Results and discussion

### TLR5 is expressed in mouse lung, liver, small intestine and kidney

**Figure 1:**
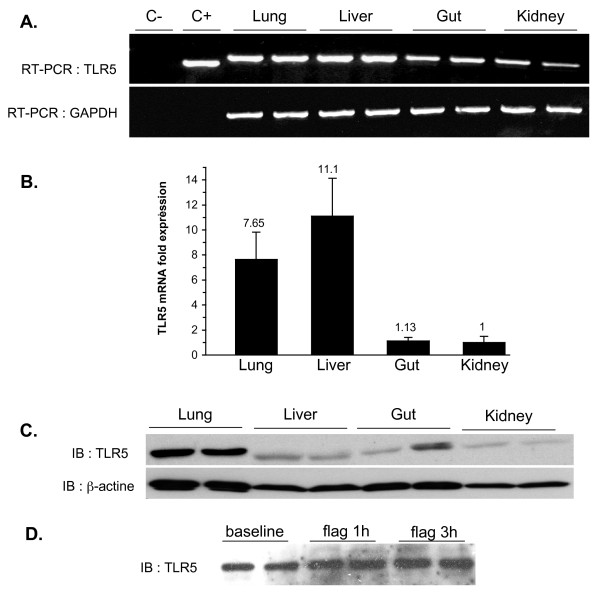
**Tissue expression of TLR5**. **(a) **Constitutive expression of TLR5 mRNA (RT-PCR) in mouse lung, liver, gut and kidney. C-, negative control (no RNA); C+, positive control of the TLR5 primer kit. Sizes of the amplified fragments were 310 base pairs for positive control and 381 base pairs for TLR5. GAPDH, loading control. **(b) **Quantitative TLR5 mRNA expression in mouse lung, liver, gut and kidney. In each organ, TLR5 mRNA was normalized to the levels of 18 S. The expression of TLR5 is shown relative to the kidney, and is indicated above each column. **(c) **TLR5 protein expression was determined by immunoblotting with β-actin shown as a loading control. **(d) **Expression of TLR5 protein (immunoblot) in the lung under baseline conditions (0 h), and one (1 h) and three (3 h) hours after the administration of flagellin (5 μg). There was no evidence of TLR5 induction upon flagellin. Results, shown in duplicates, are representative of at least three independent experiments.

The specific mammalian ligand of flagellin is TLR5 [4]. Our first aim was thus to determine the tissue expression of TLR5 in mice. As illustrated in Figure 1, both TLR5 mRNA (Figures 1a and 1b) and protein (Figure 1c) were detected in all organs evaluated, implying their ability to sense extracellular flagellin. These observations extend previous works, including our own, which identified TLR5 mRNA in various mammalian tissues [10], and TLR5 protein expression in the intestine [7] and myocardium [11]. Of note, quantitative PCR indicated that TLR5 mRNA expression was highest in the lung and liver. Such a high degree of TLR5 expression might be essential for the early detection of invading air-borne or blood-borne flagellated micro-organisms, as proposed previously [12]. Another finding was that TLR5 expression did not increase up to three hours after the administration of flagellin (as evaluated in the lung, Figure 1d), pointing to the lack of inducibility of TLR5 in the presence of its ligand.

### Flagellin diffusely activates NFκB and MAP kinase signaling in tissues

**Figure 2:**
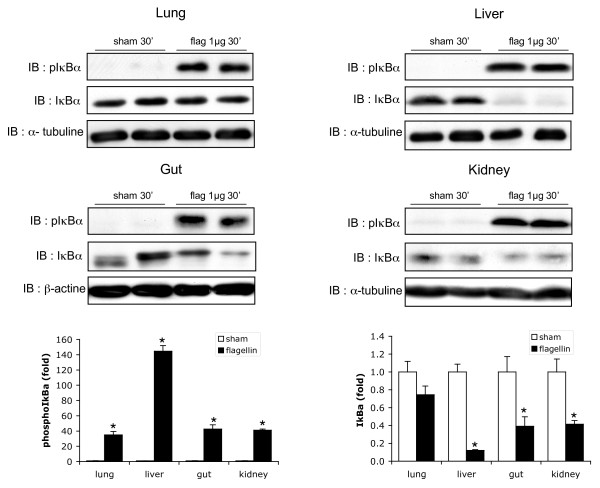
**Flagellin triggers IκBα phosphorylation and degradation in mouse organs**. Phosphorylation of inhibitor of kappaB alpha (IκBα), associated with IκBα degradation, 30 minutes after flagellin (1 μg/mouse) or saline (sham) in mouse lung, liver, gut and kidney; α-tubulin and β-actin, loading controls. Densitometric analysis, * *P *< 0.05 flagellin vs sham, t-test (*n *= 5 mice/group).

The widespread expression of TLR5 indicates that the examined organs might have the ability to mount innate immune defense mechanisms upon the recognition of circulating flagellin. It is known that TLR-dependent immune responses mostly rely on the activation of the key transcription factor NFκB, a family of dimeric transcription factors, normally held in the cytoplasm of quiescent cells, bound to an inhibitory protein, IκBα [13]. NFκB activation relies on stimulus-induced IκBα phosphorylation and proteasomal degradation, allowing free NFκB to translocate into the nucleus and to bind to its response element on the DNA [13]. As shown in Figure 2, flagellin (1 μg/mouse) induced significant IκBα phosphorylation and degradation after 30 minutes in the different tissues examined. In the lung, the degradation appeared less pronounced, probably reflecting a larger pool of total IκBα, making the degradation more difficult to detect.

**Figure 3:**
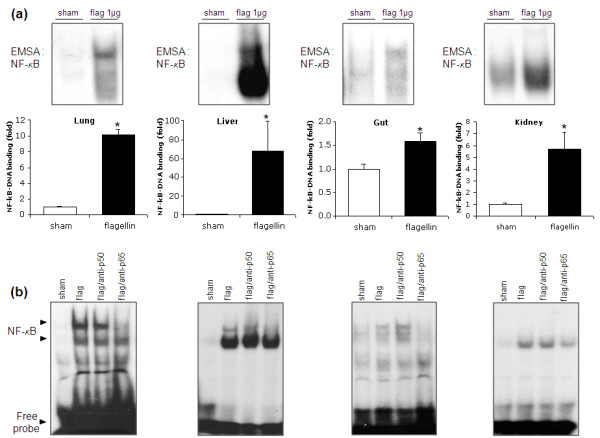
**Flagellin induces NF-κB-DNA binding in the lung, liver, gut and kidney**. **(a) **Nuclear factor (NF)-κB-DNA binding activity (electromobility shift assay (EMSA)) in mouse tissues 30 minutes after flagellin (1 μg/mouse) or saline (sham). **(b) **Supershift experiments. Nuclear proteins were incubated with an anti-p50 or an anti-p65 antibody prior to incubation with the radioactive probe. In all organs, the shifted bands supershifted in the presence of anti-p65. A large shifted band, which was not affected by anti-p65 and anti-p50 was detected in the liver. Densitometric analysis, * *P *< 0.05 flagellin vs sham, t-test (*n *= 5 mice/group).

Further evidence of NFκB activation is indicated in Figure 3, showing the results of NFκB-DNA binding experiments by EMSA. Within 30 minutes, flagellin triggered a robust increase of NFκB-DNA binding, an effect that was particularly marked in the liver, with more than a 60-fold increase compared with control conditions (Figure 3a). Interestingly, the positions of the shifted bands were different in the liver than in the other organs. As NFκB is a family of heterodimeric (and homodimeric) proteins that include p50 (and its precursor p105), p52 (and its precursor p100), p65, RelB, and c-Rel, this observation suggests that the composition of the NFκB dimer in the liver differs from the other organs. Given that the most common dimer is formed from a p50 and p65 subunit, we performed supershift experiments using anti-p50 and anti-p65 antibodies. As shown in Figure 3b, the bands were supershifted only by the anti-p65 antibody, implying that the NFκB complex contains significant amounts of p65 in all organs exposed to flagellin. The liver disclosed an additional large shifted band, which did not supershift in the presence of anti-p50 and anti-p65, further supporting a distinct hetero-or homodimeric composition of NFκB in this organ.

These findings are the first to formally demonstrate the ability of flagellin, at very low concentrations, to promote diffuse NFκB activation *in vivo*. It is noticeable that the degree of NFκB activation was the smallest in the lungs and intestines, which may indicate that organs naturally exposed to environmental micro-organisms have developed specific mechanisms to down-regulate TLR5-dependent signaling in order to prevent overwhelming and permanent inflammatory responses, an issue that should be addressed in future studies.

**Figure 4:**
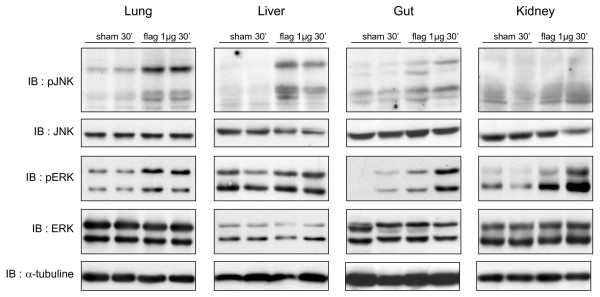
**Flagellin activates MAPK signaling pathway in mouse organs**. Mitogen-activated protein kinase (MAPK) activation, evaluated by the phosphorylation state of c-jun-NH2 terminal kinase (JNK) and extracellular related kinase (ERK), in mouse organs 30 minutes after the injection of flagellin (1 μg/mouse) or saline (sham). Phosphorylation of JNK was detected in the lung, liver, and gut, but not the kidney. ERK phosphorylation was present in the lung, gut and kidney, but not in the liver. Total JNK and ERK signals were not influenced by flagellin; α-tubulin, loading controls. Blots, shown in duplicates, are representative of at least three independent experiments.

Besides NFκB, TLRs also signal through the MAPK cascade, especially the stress-activated protein kinase JNK and ERK [14]. Figure 4 shows that flagellin promoted strong phosphorylation of JNK in the lung and liver, contrasting with a weak activation in the gut and kidney. With respect to ERK, its phosphorylation was enhanced in the lung, gut and kidney, and less in the liver, in which ERK was already phosphorylated under baseline conditions. These novel observations point to significant organ specificity in the modulation of TLR5-dependent signaling in response to flagellin, a feature also observed with other bacterial components triggering innate immune responses, such as peptidoglycan [15]. The mechanisms underlying such organ-specific responses cannot be determined from our current data and thus will need further investigations.

**Figure 5:**
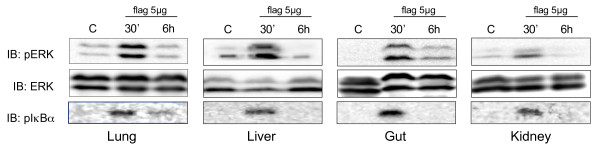
**Transient nature of mitogen-activated protein kinase and nuclear factor-κB activation in response to flagellin**. The phosphorylation state of extracellular related kinase (ERK) and inhibitor of kappaB alpha (IκBα) was evaluated by immunoblotting in organs from mice under baseline conditions (Control C) and from mice challenged with 5 μg of flagellin for 30 minutes or 6 hours. The phosphorylation noted at 30 minutes did not persist after 6 hours. Blots are representative of three independent experiments.

It is worth mentioning that an increase in the dose of flagellin to 5 μg/mouse did not exert any further increase in the activation of MAPKs and NFκB, as shown by a comparable degree of ERK and IκBα phosphorylation in the different organs (Figure 5), suggesting that minute doses of flagellin are already sufficient to produce a maximal activation of these stress signaling cascades. This observation is consistent with the marked pro-inflammatory potency of flagellin, which has been shown to induce a pro-inflammatory response in the lung at a threshold of 10 ng/mouse [6]. A further finding was the transient nature of the process of immune activation triggered by flagellin, as indicated by the absence of sustained phosphorylation of ERK and IκBα six hours after the injection of flagellin (Figure 5). This time-course is indeed typical of the usual short-lived pattern of MAPKs and NFκB activation due to the rapid induction of negative feedback regulatory mechanisms [5,16-18]. Such balance between immune activation and subsequent deactivation is critical to eliminate invading pathogens, while at the same time restraining the risk of self-destruction by unopposed inflammatory responses [18].

### Flagellin promotes widespread generation of inflammatory cytokines

**Figure 6:**
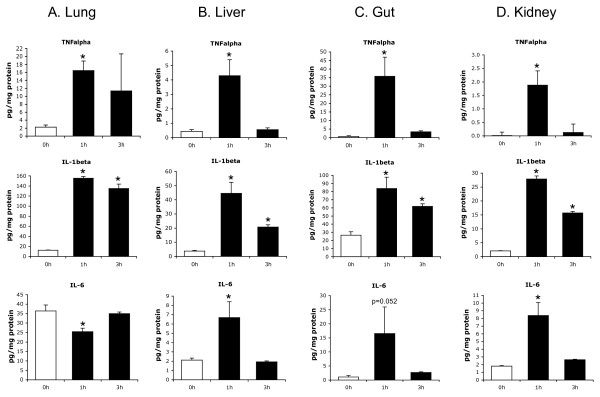
**Flagellin triggers the diffuse expression of pro-inflammatory cytokines**. Levels of inflammatory cytokines, measured by ELISA, in **(a) **lung, **(b) **liver, **(c) **gut and **(d) **kidney, at baseline and one and three hours after flagellin administration (5 μg/mouse). * *P *< 0.05 versus baseline (analysis of variance followed by Dunnett's test, *n *= 5 to 6 mice/group).

**Figure 7:**
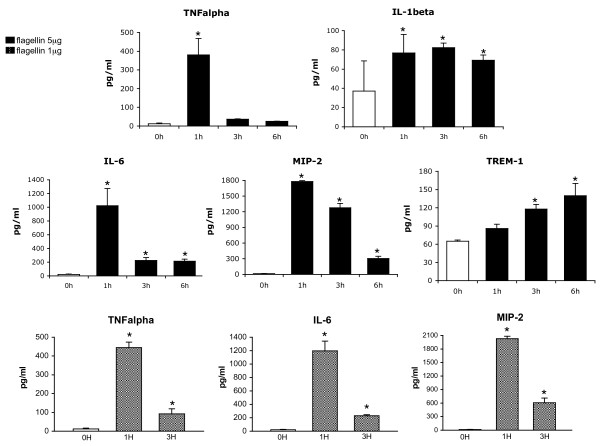
**Time-course of plasma cytokines after flagellin administration**. Cytokines were quantified by ELISA in plasma at baseline (time 0), at one, three and six hours after 5 μg flagellin, and at one and three hours after 1 μg flagellin in separate groups of mice (*n *= 5 to 6 mice/group). An early peak (1 h) was noted for TNFα, IL-6 and macrophage-inhibitory protein-2 (MIP-2), whereas IL-1β and soluble triggering receptor expressed on myeloid cells (sTREM-1) steadily increased over six hours. * *P *< 0.05 versus baseline (analysis of variance followed by Dunnett's test).

The activation of TLRs ultimately results in the enhanced expression of multiple inflammatory mediators [19], which stand at the foreground of the systemic inflammation and multiple organ dysfunction characterizing septic shock [20]. Accordingly, the activation of innate immune signaling by flagellin translated into the establishment of a pro-inflammatory phenotype in the organs examined, as shown by the up-regulated expression of the cytokines TNFα, IL-1β, IL-6, sTREM-1, and the chemokine MIP-2 (the rodent equivalent of human IL-8) in these organs (Figure 6), as well as in plasma (Figure 7), after flagellin administration. Similarly to the activation of NFκB and ERK discussed above, the rise in plasma cytokines (at least for TNFα, IL-6 and MIP-2) was similar at 1 or 5 μg, further supporting the considerable pro-inflammatory potency of this bacterial protein. In spite of this diffuse inflammatory response, it is worth mentioning that the doses of flagellin used in this study were not lethal, as indicated by the lack of any mortality observed up to 48 hours after flagellin administration.

The production of cytokines followed two distinct time-courses. First, TNFα, IL-6 and MIP-2 in tissues showed an early peak after one hour and a steady decline thereafter, with a return to baseline concentrations at three hours (for TNFα and IL-6). A noticeable exception was the lung, in which IL-6 did not show any increase over time after flagellin, but it is noteworthy that the concentration of IL-6 was already elevated under baseline conditions in this organ, an observation already reported by others [21,22]. In plasma, TNFα increased only transiently, with a peak at one hour followed by return to baseline values at later time-points. IL-6 and MIP-2 also peaked after one hour, but were still significantly elevated at three and six hours. Such profile is comparable with the hyperacute cytokine response, which is observed following the administration of LPS both in animals [23] and humans [24].

A second, distinct time-course was observed for IL-1β and sTREM-1, which remained elevated after three hours in the organs and were still significantly increased in the plasma six hours after the flagellin challenge. TREM-1, expressed at the surface of neutrophils and a subset of monocytes [25], is released as a soluble form in the plasma during sepsis and endotoxemia with a time-course similar to the one noted in the current study [26]. With respect to IL-1β, it must be underscored that its expression does not rely on NF-κB signaling, but instead on the activation of caspase-1 and inflammasome-dependent processing of pro-IL-1β [27]. Inflammasomes are high-molecular weight, caspase-1-activating platforms that control maturation and secretion of IL-1β, which are assembled upon activation of intracellular receptors by various microbial and host-derived danger signals. These receptors belong to the nucleotide binding oligomerization domain (NOD)-like receptor family, consisting of the NODs, the NOD-like receptor proteins (NLRPs) and the interleukin-1-converting enzyme protease-activating factor (IPAF) subfamilies. The IPAF inflammasome is activated by cytosolic flagellin (in contrast to the recognition of extracellular flagellin by TLR5), which is injected into the cytosol via bacterial type III and IV secretion systems. The increased expression of IL-1β observed in our study is therefore consistent with the activation of the IPAF inflammasome by flagellin [28-30]. In summary, the time-course of the different cytokines reported in the current study support the concept that flagellin triggers an early, diffuse and transient pro-inflammatory response, that is followed by the activation of mononuclear cells, promoting a long-lasting secondary response characterized by persistent elevation of sTREM-1 and IL-1β.

### Flagellin differentially triggers apoptotic signaling in mouse organs

Multiple organ dysfunction in sepsis generally occurs in the absence of significant apoptosis or necrosis in the failing organs, except from the gut, in which extensive apoptosis of epithelial cells has been reported [31]. Apoptosis in the gut may be particularly detrimental in sepsis by compromising bowel wall integrity, as suggested by studies reporting significantly reduced sepsis mortality in transgenic mice overexpressing the antiapoptotic protein Bcl-2 in their intestinal epithelium [32,33]. Here we sought to determine the ability of flagellin to initiate apoptotic signaling in mouse organs, by determining the degree of activation of capase-3, one of the major executioner caspases, and the cleavage of its substrate PARP [34].

**Figure 8:**
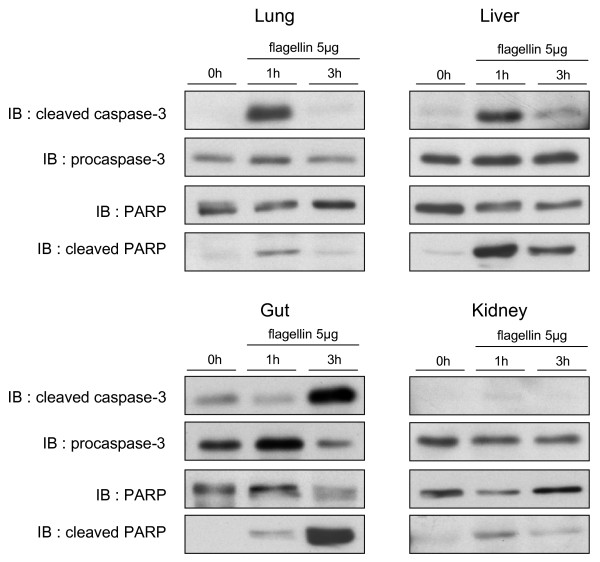
**Flagellin activates pro-apoptotic signaling in mouse tissues**. Cleavage of caspase-3 and poly(ADP-ribose) polymerase (PARP; immunoblotting) in mouse organs at baseline (time 0), and at one and three hours after flagellin (5 μg). Caspase-3 and PARP were cleaved slightly at one hour in the liver and lung, and strongly at three hours in the gut. No cleavage of caspase-3 was noted in the kidney, which disclosed a discrete cleavage of PARP at one hour. Procaspase-3 and uncleaved PARP did not change over time, except in the gut where both decreased after three hours. Blots are representative of at least three independent experiments.

As illustrated in Figure 8, flagellin triggered only transient caspase-3 and PARP cleavage in the lung and liver, pointing to limited apoptosis and rapid clearance of apoptotic cells in these organs. In the kidney, except from a slight cleavage of PARP at one hour, apoptotic changes were virtually absent. These observations are totally consistent with the low degree of apoptosis reported in the liver, lung and kidney during sepsis [35]. In marked contrast, significant apoptotic changes were present in the gut, as indicated by a very strong cleavage of both caspase-3 and PARP, three hours after flagellin administration. It is particularly noteworthy that a previous study indicated that apoptosis within the gut during polymicrobial sepsis is endotoxin-independent [36]. Thus, our findings suggest that flagellin might represent an important culprit of these changes in this setting.

### Study limitations

The different measurements in our study were performed at the level of whole organs containing multiple cell populations (parenchymal, vascular and inflammatory cells). Therefore, we cannot comment further on the respective implication of each of these cell types in the observed effects of flagellin. Further studies using histochemical labeling would be necessary to address this issue. A second limitation is the lack of direct comparisons between the effects of flagellin and endotoxin in our study. However, due to the extensive documentation of the effects of endotoxin in the murine model, we opted to focus primarily on the systemic effects of flagellin, which have remained so far largely unexplored. Thirdly, with respect to apoptosis, we only relied on molecular markers and thus, we cannot provide direct quantification of the severity of apoptosis in the different organs evaluated, which would require further immunohistochemical studies.

## Conclusions

In conclusion, the data presented herein are the first to formally demonstrate that TLR5 is widely expressed in mouse organs *in vivo*, that flagellin elicits prototypical innate immune responses in these organs, and that it also activates apoptotic signaling, predominantly in the intestine. These findings imply that flagellin may be instrumental in the pathogenesis of Gram-negative sepsis. Hence, the therapeutic potential of anti-flagellin or anti-TLR5 strategies in this setting should be tested in the future.

## Key messages

• TLR5, the specific mammalian receptor for bacterial flagellin, is expressed in the lung, kidney, liver and small intestine of mice, both at the mRNA and protein level.

• Recombinant *Salmonella *flagellin elicits widespread innate immune responses in mouse organs, characterized by the activation of NF-κB and MAPKs.

•The innate immune response to bacterial flagellin promotes systemic inflammation and triggers apoptotic signalling in the intestine.

• Flagellin/TLR5 signaling may be instrumental in the pathogenesis of Gram-negative sepsis.

## Abbreviations

ELISA: enzyme-linked immunosorbent assay; EMSA: electromobility shift assay; ERK: extracellular related kinase; IκBα: inhibitor of kappaB alpha; IL: interleukin; JNK: c-jun-NH2 terminal kinase; LPS: lipopolysaccharide; MAPK: mitogen-activated protein kinase; MIP: macrophage-inhibitory protein; NF-κB: nuclear factor kappaB; NLRP: NOD-like receptor protein; NOD: nucleotide binding oligomerization domain; IPAF: interleukin-1-converting enzyme protease-activating factor; PARP: poly(ADP-ribose) polymerase; PCR: polymerase chain reaction; sTREM-1: soluble triggering receptor expressed on myeloid cells; TLR: toll-like receptor; TNFα: tumor necrosis alpha.

## Competing interests

The authors declare that they have no competing interests.

## Authors' contributions

JR, NL, SL, NRV, and SRC performed the experimental work. LL, PP, and NRV contributed to the conception and design of the study. LL, FF, BW, and PP contributed to analysis of data, manuscript drafting and revising. LL obtained funding.
